# Exploring the relationship between life course adiposity and sepsis: insights from a two-sample Mendelian randomization analysis

**DOI:** 10.3389/fendo.2024.1413690

**Published:** 2024-06-14

**Authors:** Zimei Cheng, Jingjing Li, Wenjia Tong, Tingyan Liu, Caiyan Zhang, Jian Ma, Guoping Lu

**Affiliations:** ^1^ Department of Pediatric Intensive Care Unit, Children’s Hospital of Fudan University, National Children’s Medical Center, Shanghai, China; ^2^ Shanghai Institute of Infectious Disease and Biosecurity, Fudan University, Shanghai, China; ^3^ Department of Pediatric Intensive Care Unit, Anhui Provincial Children's Hospital, Hefei, China

**Keywords:** obesity paradox, pediatric obesity, body mass index, obesity, abdominal, waist circumference, fat body

## Abstract

**Objectives:**

The relationship between adiposity and sepsis has received increasing attention. This study aims to explore the causal relationship between life course adiposity and the sepsis incidence.

**Methods:**

Mendelian randomization (MR) method was employed in this study. Instrumental variants were obtained from genome-wide association studies for life course adiposity, including birth weight, childhood body mass index (BMI), childhood obesity, adult BMI, waist circumference, visceral adiposity, and body fat percentage. A meta-analysis of genome-wide association studies for sepsis including 10,154 cases and 454,764 controls was used in this study. MR analyses were performed using inverse variance weighted, MR Egger regression, weighted median, weighted mode, and simple mode. Instrumental variables were identified as significant single nucleotide polymorphisms at the genome-wide significance level (*P* < 5×10^-8^). The sensitivity analysis was conducted to assess the reliability of the MR estimates.

**Results:**

Analysis using the MR analysis of inverse variance weighted method revealed that genetic predisposition to increased childhood BMI (*OR* = 1.29, *P* = 0.003), childhood obesity (*OR* = 1.07, *P* = 0.034), adult BMI (*OR* = 1.38, *P* < 0.001), adult waist circumference (*OR* = 1.01, *P* = 0.028), and adult visceral adiposity (*OR* = 1.53, *P* < 0.001) predicted a higher risk of sepsis. Sensitivity analysis did not identify any bias in the MR results.

**Conclusion:**

The results demonstrated that adiposity in childhood and adults had causal effects on sepsis incidence. However, more well-designed studies are still needed to validate their association.

## Introduction

Sepsis, defined as life-threatening organ dysfunction resulting from a dysregulated host response to infection, poses a major healthcare challenge (1). There are more than 48 million reported cases of sepsis worldwide annually, accompanied by a mortality rate ranging from 22.5% to 35.0%, contributing to approximately one in five of all global deaths (2–4). And the survivors of sepsis often experience post-intensive care syndrome and chronic physical disabilities. Although infection (including bacteria, fungi, viruses, etc.) is the direct cause of sepsis, many factors such as diabetes, hypertension, neurological disorders, obesity, etc., also contribute to an increased risk of sepsis (5–7). Due to the rapid onset and progression of sepsis, primary prevention targeting the risk factors for sepsis becomes even more important.

Adiposity, characterized by being overweight or obese, has emerged as a global health problem. Current estimates suggest a global obesity prevalence of about 38%, expected to rise to 51% by 2035 (8). Adiposity, has been proven to be associated with cardiovascular diseases, diabetes, cancer, and other conditions (9–11). A survey indicates that up to 60% of patients with sepsis in the ICU are overweight or obese (12). In recent years, the impact of obesity on sepsis has increasingly drawn researchers’ interest. On the one hand, some observational studies have found that obesity is associated with an increased risk of infection, including COVID-19, severe influenza, and surgical site infections (13–15). On the other hand, other studies have found that patients with sepsis who are obese have relatively higher survival rates (16–20). Additionally, population-based studies have reported increased overall mortality due to sepsis in obese individuals (21, 22). These seemingly contradictory findings highlight the complex impact of obesity on sepsis under different pathophysiological conditions. Consequently, further research is imperative to elucidate the relationship between obesity and sepsis, offering fresh perspectives and insights for the prevention and treatment of this condition.

Currently, there are several issues with research on obesity and sepsis. Firstly, most studies on these two conditions are observational, which are often affected by potential biases, confounding factors, and reverse causality, leading to unreliable conclusions. And ethical concerns prevent the conduct of randomized controlled trials to definitively establish the impact of obesity on sepsis. Secondly, many studies currently use BMI as the indicator of obesity. However, besides BMI, the degree of adiposity can also be measured by waist circumference (WC), visceral adiposity, body fat percentage (BFP), and other indicators. Finally, but most importantly, the literature often relies on cross-sectional data at the time of onset of illness, while obesity status can change over time. The issue of adiposity transcends various life stages. Studying the relationship between adiposity during the life course and sepsis incidence helps to understand underlying mechanisms and formulate preventive measures.

Due to ethical constraints, conducting randomized controlled trials (RCTs) to study the relationship between adiposity and sepsis incidence is limited. This research employed a two-sample Mendelian randomization (MR) study and aimed to identify the causal effects of life course adiposity on sepsis incidence. The MR design method leverages publicly available datasets of risk factors and diseases obtained from comprehensive genome-wide association studies (GWAS) to assess relationship between exposures and outcomes. As an epidemiological approach, MR studies utilize single nucleotide polymorphisms (SNPs) randomly assigned during conception as an instrumental variable, offering a robust method for inferring causal relationships similar to randomized controlled trials (RCTs) (23). This approach helps mitigate the influence of confounding factors and reverse causality, providing valuable insights into the nuanced relationship between obesity indicators and sepsis risk across the life cycle.

## Materials and methods

### Study design

**Figure 1:**
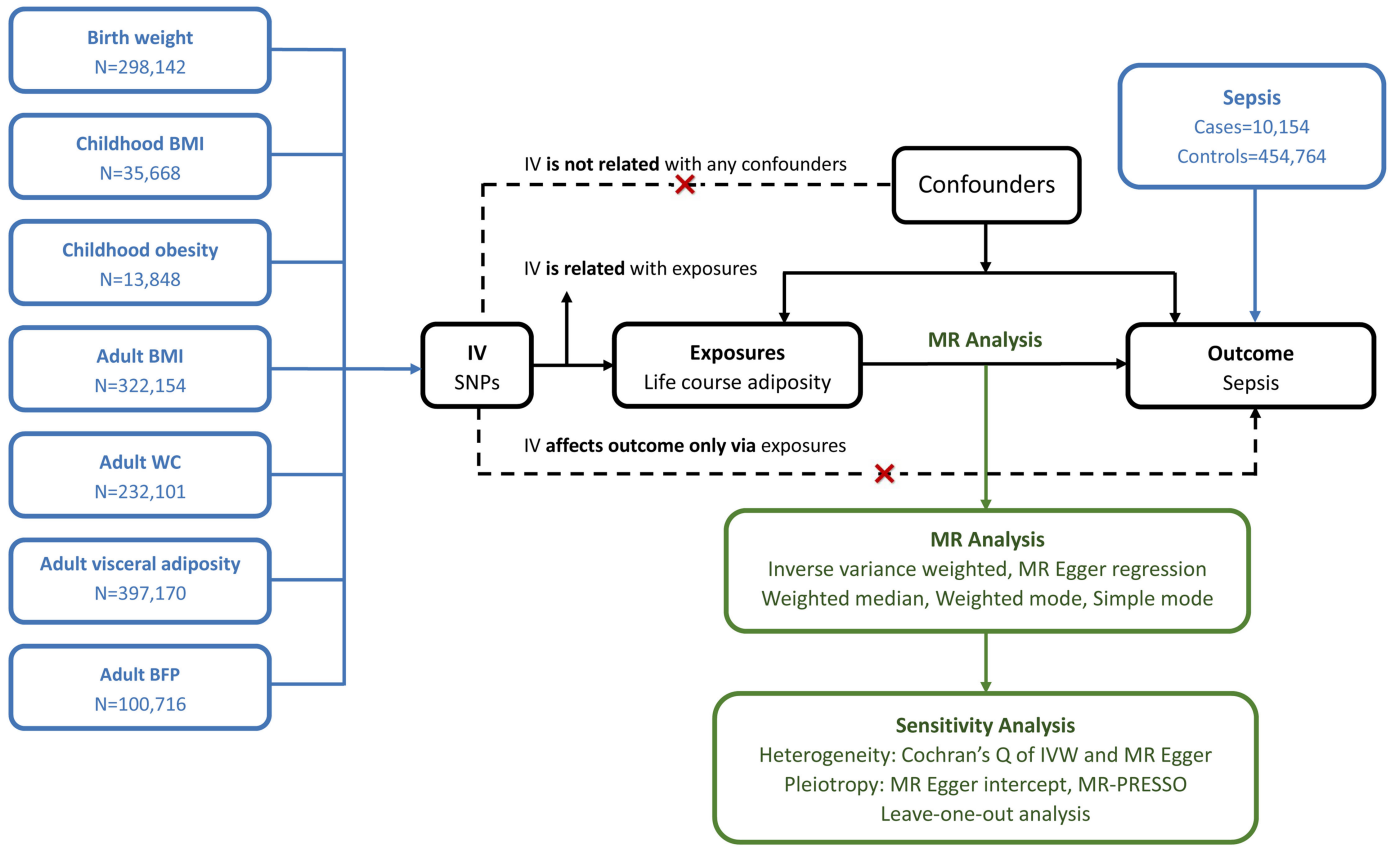
Principles of Mendelian randomization. BMI, body mass index; WC, waist circumference; BFP, body fat percentage; IV, inverse variance; MR, Mendelian randomization; IVW, inverse variance weighted; MR-PRESSO, Mendelian randomization pleiotropy residual sum and outline.

We conducted a two-sample MR study to investigate the causal relationship between life course adiposity and sepsis. Life course adiposity was categorized into three stages: newborns, childhood, and adults. Corresponding genetic traits from published GWAS were chosen for each stage: birth weight (BW) for the newborn stage; childhood BMI and childhood obesity for the child stage; adult BMI, adult WC, adult visceral adiposity, and adult BFP for the adult stage. The overview of the study design is shown in **Figure 1**. The Mendelian randomization design relies on three fundamental assumptions: (1) genetic variation exhibits a strong association with the exposure of interest; (2) genetic variation remains independent of any other potential confounding variables; (3) genetic variation solely influences the outcome through its impact on the investigated exposure. As our study relied on publicly available databases and previously published studies, no additional ethics approval was required.

### Data sources

We conducted a search for GWASs related to these traits and identified eight recently published datasets characterized by large sample sizes and comprehensive information on genetic variants. The basic characteristics of these GWASs are described in **Table 1**.

**Table 1:** Basic characteristics of included GWAS of birth weight, childhood BMI, childhood obesity, adult BMI, adult WC, adult visceral adiposity, adult BFP, and sepsis.

Exposure/Outcome	Consortium/First author	Sample size	Ancestry	Web source/Pubmed ID
Birth Weight	Early Growth Genetics Consortium	298142	European ancestry	https://egg-consortium.org/
Childhood BMI	Felix JF	35668	European ancestry	26604143
Childhood obesity	Early Growth Genetics Consortium	24160	European ancestry	https://egg-consortium.org/
Adult BMI	Locke AE	322154	European ancestry	25673413
Adult WC	Shungin D	232101	European ancestry	25673412
Adult visceral adiposity	Torgny Karlsson	397170	European ancestry	31501611
Adult BFP	Lu YC	100716	88.7% of European ancestry	26833246
Sepsis	Hamilton F	486484	European ancestry	UKB consortium

GWAS, genome-wide association studies; BMI, body mass index; WC, waist circumference; BFP, body fat percentage.

The genetic variants associated with BW were extracted from the Early Growth Genetics Consortium, which involved 298,142 individuals of European descent (24). Birth weight, defined as the weight of a neonate measured within the first hours of life, is recorded immediately after birth and before any substantial postnatal weight loss occurs.

For childhood BMI, genetic variants were extracted from a GWAS meta-analysis involving 35,668 children of European ancestry aged between 2 and 10 years (25). BMI, a numerical value derived from an individual’s height and weight, is calculated as the individual’s mass in kilograms divided by the square of their height in meters (kg/m²).

The genetic variants associated with childhood obesity were obtained from the Early Growth Genetics Consortium, involving 13,848 children aged between 2 and 18 years, with childhood obesity defined as being at or above the 95th percentile of BMI (26).

Regarding adulthood phenotypes, genetic variants for adult BMI, adult WC, adult visceral adiposity, and adult BFP were obtained from several GWAS meta-analysis. The genetic variants for adult BMI were sourced from a GWAS and Metabochip meta-analysis conducted by Locke AE et al., involving a total of 322,154 European individuals (27).

The genetic variants for adult WC were drawn from a genome-wide association meta-analyses that included 232,101 individuals (28). The WC refers to a direct anthropometric measurement representing the distance around the smallest area below the rib cage and above the iliac crest, measured at the level of the navel.

Data on visceral adiposity were obtained from a GWAS performed by Torgny Karlsson et al. with 397,170 European individuals, and visceral adiposity levels were estimated using prediction models (29).

The genetic variants for adult BFP were extracted from a genome-wide association meta-analysis involving 100,716 individuals from 56 studies, with 88.7% of the participants having European ancestry. The BFP in each study was measured either with bioimpedance analysis or dual energy X-ray absorptiometry (30).

The GWAS data of sepsis were obtained from the UKB consortium, which included 10154 sepsis cases and 454764 controls of European ancestry. Explicit sepsis is defined in our study referring to a previously published list International Classification of Disease-9 and Disease-10 codes derived by a panel of experts in critical care, infectious diseases, and sepsis epidemiology.

### Selection of instrumental variables

Our study employs following criteria for the selection of instrumental variables: (1) Significant SNPs associated with BW, childhood BMI, childhood obesity, adult BMI, adult WC, adult visceral adiposity, and adult BFP at the genome-wide significance level (*P* < 5×10^-8^) were identified as instrumental variables in our study. These SNPs were further refined by pruning at linkage disequilibrium with an *r^2^* threshold of <0.001 within a 10Mb distance. (2) The strength of chosen instrumental variables was evaluated using F-statistics (*F* > 10) to mitigate potential bias arising from weak instruments. F-statistics were computed using the formula *R²* × (*n* - *k* - 1)/*k* (1 - *R²*), where *n*, *k*, and *R²* represent the sample size, the number of SNPs, and the variance explained by the instrumental variables, respectively. (3) SNPs that were disqualified from the MR analysis during the harmonization process due to being palindromic or containing incompatible alleles were systematically excluded. This meticulous step was taken to ensure the concordance of effect alleles. (4) The selected instrumental variables were verified to be non-associated with both the outcome variable and any potential confounders, ensuring the robustness of the instrument selection.

### Statistical analysis

To estimate the causal effect of life course adiposity on sepsis, we utilized the aforementioned SNPs for MR analysis. Our study employed five distinct MR analysis methods: inverse variance weighted (IVW), MR Egger regression, weighted median, weighted mode, and simple mode. In the IVW meta-analysis approach, the outcome effects of instrumental variables on exposure effects are transformed into a weighted regression, with a fixed intercept at zero. This technique relies on the assumption of the absence of horizontal pleiotropy and seeks to produce unbiased estimates by minimizing the impact of confounding factors. MR Egger accounts for potential pleiotropy in estimating causal effects by utilizing an intercept term. By assigning weights to SNPs, the weighted median method provides a robust estimate even when up to 50% of the weight comes from invalid instruments. The weighted mode method demonstrates adaptability in cases where the genetic variable challenges the pleiotropy hypothesis. Similar to the weighted mode, the simple mode method may offer robustness against pleiotropy. The simple mode method identifies the mode of the distribution but assigns equal weight to each estimate. This comprehensive approach enhances the reliability of our findings.

We performed a sensitivity analysis of the MR results. The heterogeneity between instrumental variables was analyzed by Cochran’s Q test, including IVW and MR Egger. We utilized IVW as the major approach, which combined Wald ratios across the exposure -associated SNPs to get a summary estimate. MR Egger intercept and Mendelian randomization pleiotropy residual sum and outlier (MR-PRESSO) were performed to evaluate horizontal pleiotropy. In the final stage, we executed a leave-one-out analysis to meticulously assess the impact of individual SNPs on MR estimates.

The results are displayed in the form of odds ratios (*ORs*) and 95% confidence intervals (*CIs*). A Bonferroni-corrected threshold calculated as 0.007 (0.05/7 exposure phenotypes) was employed to address multiple comparisons. *P*-values lower than 0.007 can indicate a confirmed causal link. *P*-values between 0.007 and 0.05 were considered indicative proof of an underlying association. *P*-values greater than 0.05 implied a lack of causal relationship. All analyses were conducted using the “TwoSampleMR” package in R software (version 4.2.1).

## Results

### MR estimate of life course adiposity on sepsis

After quality control, 128 SNPs for birth weight, 14 SNPs for childhood BMI, 14 SNPs for childhood obesity, 53 SNPs for adult BMI, 31 SNPs for adult WC, 175 SNPs for adult visceral adiposity and 7 SNPs for adult BFP were confirmed for MR analysis. All instrumental variables with their beta effects and standard errors (SE) associated with life course adiposity and sepsis were listed in **Supplementary Table S1**.

**Figure 2:**
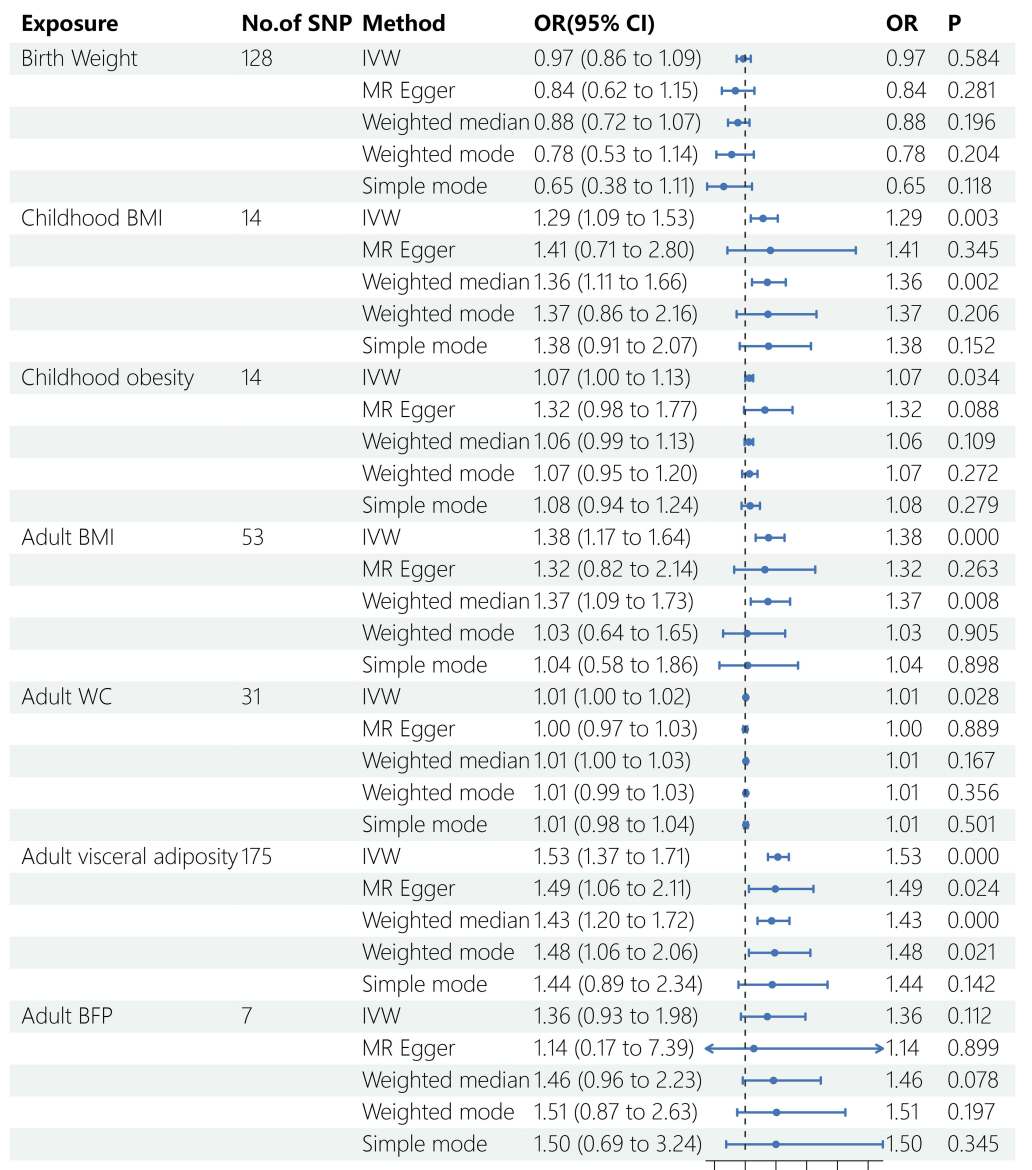
Results of MR analysis. SNP, single nucleotide polymorphisms; OR, odds ratio; BMI, body mass index; WC, waist circumference; BFP, body fat percentage; IVW, inverse variance weighted; MR, Mendelian randomization.

**Figure 3:**
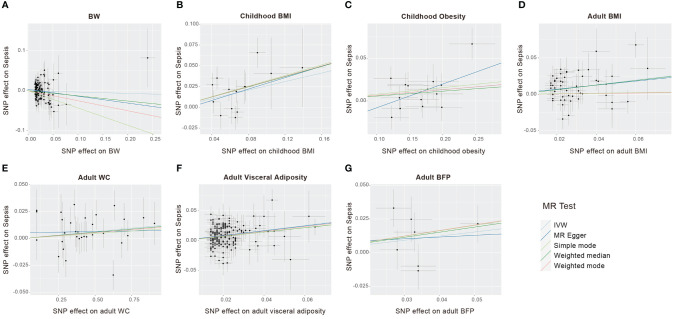
Scatter plots to visualize causal effect of life course adiposity [**(A)** BW; **(B)** Children BMI; **(C)** Children Obesity; **(D)** Adult BMI; **(E)** Adult WC; **(F)** Adult Visceral Adiposity; **(G)** Adult BFP] on the risk of sepsis.

Five Mendelian randomization methods, including IVW, MR Egger regression, weighted median, weighted mode, and simple mode, were employed to investigate the causal effects of life course adiposity on the risk of sepsis. Analysis using the IVW method revealed that genetic predisposition to increased childhood BMI (*OR* = 1.29, 95% *CI* = 1.09–1.53, *P* = 0.003), childhood obesity (*OR* = 1.07, 95% *CI* = 1.00–1.13, *P* = 0.034), adult BMI (*OR* = 1.38, 95% *CI* = 1.17–1.64, *P* < 0.001), adult WC (*OR*= 1.01, 95% *CI* = 1.00–1.02, *P* = 0.028), and adult visceral adiposity (*OR* = 1.53, 95% *CI* = 1.37–1.71, *P* < 0.001) predicted a higher risk of sepsis. However, there was no evidence of causal effects for BW (*OR* = 0.76, 95% *CI* = 0.86–1.09, *P* = 0.584) and adult BFP (*OR* = 1.36, 95% *CI* = 0.93–1.98, *P* = 0.112) on sepsis. Similar direction and magnitude of causal inference between BW, childhood BMI, childhood obesity, adult BMI, adult WC, adult visceral adiposity, adult BFP and sepsis were observed using the MR-Egger, weighted median model, weighted mode, and simple mode analysis, reinforcing the reliability of our results (**Figures 2**, **3**, **Table 2**).

**Table 2:** Causal estimations of life course adiposity on sepsis.

Life course adiposity	IVW			MR Egger			Weighted median			Weighted mode			Simple mode		
	OR	95%CI	P	OR	95%CI	P	OR	95%CI	P	OR	95%CI	P	OR	95%CI	P
Birth Weight	0.97	0.86-1.09	0.584	0.84	0.62-1.15	0.281	0.88	0.72-1.07	0.196	0.78	0.53-1.14	0.204	0.65	0.38-1.11	0.118
Childhood BMI	1.29	1.09-1.53	**0.003**	1.42	0.71-2.80	0.345	1.36	1.11-1.66	0.002	1.37	0.86-2.16	0.206	1.38	0.91-2.07	0.152
Childhood obesity	1.07	1.00-1.13	**0.034**	1.32	0.98-1.77	0.088	1.06	0.99-1.14	0.116	1.07	0.94-1.21	0.314	1.08	0.94-1.24	0.279
Adult BMI	1.38	1.17-1.64	**0.00015**	1.32	0.82-2.14	0.263	1.37	1.09-1.73	0.008	1.03	0.64-1.65	0.905	1.04	0.58-1.86	0.898
Adult WC	1.01	1.00-1.02	**0.028**	1.00	0.97-1.03	0.889	1.01	1.00-1.03	0.167	1.01	0.99-1.03	0.356	1.01	0.98-1.04	0.501
Adult visceral adiposity	1.53	1.37-1.71	**9.62E-14**	1.49	1.06-2.11	0.024	1.43	1.20-1.72	8.22E-05	1.48	1.06-2.06	0.021	1.44	0.89-2.34	0.142
Adult BFP	1.36	0.93-1.98	0.112	1.14	0.17-7.39	0.899	1.46	0.95-2.23	0.081	1.51	0.90-2.53	0.170	1.50	0.69-3.24	0.345

IVW, inverse variance weighted; MR, Mendelian randomization; OR, odds ratio; CI, confidence interval; BMI, body mass index; WC, waist circumference; BFP, body fat percentage.

The bold values means *P* < 0.05.

### Sensitivity analysis of MR estimates

**Figure 4:**
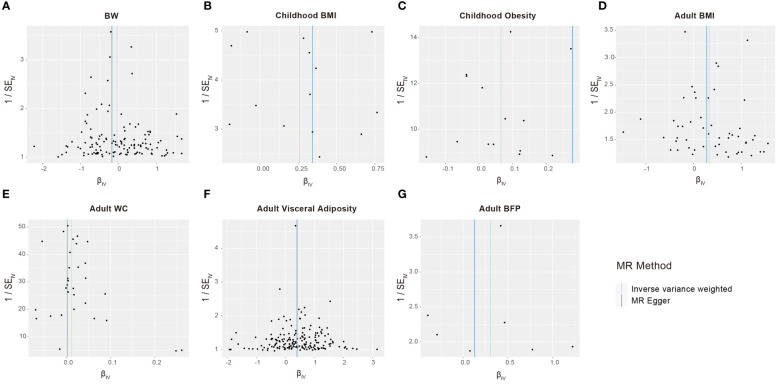
Funnel plots to visualize overall heterogeneity of Mendelian randomization estimates for the effect of life course adiposity [**(A)** BW; **(B)** Children BMI; **(C)** Children Obesity; **(D)** Adult BMI; **(E)** Adult WC; **(F)** Adult Visceral Adiposity; **(G)** Adult BFP] on the risk of sepsis.

We conducted a sensitivity analysis to identify potential biases in our analysis. The *F*-statistics for all selected SNPs were consistently larger than 10, suggesting the absence of weak instrumental variable bias (**Supplementary Table S1**). Cochran’s Q test applied to both the MR-Egger and IVW approaches revealed no significant heterogeneity among the selected SNPs (**Table 3**). The results of the funnel chart also support this conclusion (**Figure 4**). All *P*-values for the MR Egger intercept and MR-PRESSO global test exceeded 0.05, indicating the absence of directional horizontal pleiotropy (**Table 3**). The findings highlight the robustness of the identified causal effects from life course adiposity to sepsis, as the results remained consistent in direction. Additionally, leave-one-out analysis and single SNP analysis did not reveal any notable abnormalities (**Supplementary Figure S1**). These results collectively underscore the reliability and robustness of our study.

**Table 3:** Heterogeneity and directional pleiotropy analyses results using different methods.

Life course adiposity	Heterogeneity		Horizontal pleiotropy	
	Q_P value (IVW)	Q_P value (MR Egger)	MR Egger Intercept P	MR-PRESSO global test P
BW	125.96_0.51	125.07_0.51	0.347	0.486
Childhood BMI	20.61_0.08	20.51_0.06	0.803	0.090
Childhood obesity	20.09_0.929	17.09_0.146	0.173	0.095
Adult BMI	65.66_0.10	65.61_0.08	0.841	0.089
Adult WC	30.81_0.42	30.26_0.40	0.473	0.410
Adult visceral adiposity	181.79_0.33	181.78_0.31	0.898	0.334
Adult BFP	8.81_0.18	8.74_0.12	0.855	0.250

Q, Cochran’s Q; IVW, inverse variance weighted; MR, Mendelian randomization; MR-PRESSO, Mendelian randomization pleiotropy residual sum and outline; BMI, body mass index; WC, waist circumference; BFP, body fat percentage.

## Discussion

Sepsis is a significant disease that leads to health and economic losses. With the worrying rise in the prevalence of obesity in recent years, there has been growing attention on the relationship between obesity and sepsis. Presently, there exists controversy surrounding this relationship. While obesity may increase the incidence of sepsis, it also appears to be a protective factor against mortality in sepsis patients, a phenomenon known as the obesity paradox (16, 31). The underlying mechanisms of this paradox are not fully understood and may involve factors such as metabolic reserves and immune responses. Additionally, methodological limitations such as study heterogeneity, selection bias, and insufficient adjustment for confounding variables may also contribute to this phenomenon. Higher-level evidence is needed to elucidate the relationship between obesity and sepsis. This study investigates the impact of obesity throughout the life course on the incidence of sepsis. Due to ethical issues preventing the conduct of RCTs, we employed MR analysis, a method similar to RCTs.

To our knowledge, this study was the first MR analysis to meticulously evaluate the potential causal association between life course adiposity and sepsis incidence. The outcomes of our MR analysis unveiled significant associations, indicating that genetically predicted higher childhood BMI and childhood obesity, as well as elevated adult BMI, adult WC, and adult visceral adiposity, are linked to an increased risk of sepsis. Notably, we have done for the first time to discover the association between childhood adiposity and increased risk of future sepsis.

Observational studies exploring the correlation between BW and susceptibility to non-neonatal sepsis are notably scarce. BW, a valuable indicator of fetal intrauterine growth, has been linked to the onset of conditions such as insulin resistance, metabolic syndrome. During fetal intrauterine growth and development, the influence of early embryonic imprinted genes and epigenetic changes may extend to subsequent developmental stages, potentially shaping lifelong health events. However, our analysis revealed that genetically predicted BW is not significantly associated with the risk of sepsis. Several factors may contribute to this lack of association. BW is influenced by a myriad of factors, including gestational age, the number of fetuses, and maternal health, among others. Momoko et al. found that only a fraction, approximately 15%, of the variance in birth weight can be attributed to fetal genetic variation (32). Additionally, it is conceivable that both high and low birth weight could be linked to an increased risk of sepsis. The two variables may exhibit a “U-shaped” relationship. Thus, the intricate relationship between birth weight and the subsequent development of sepsis necessitates further exploration.

The prevalence of obesity among children has seen a significant rise (33). To our knowledge, this is the first study investigating the association between obesity in childhood and subsequent sepsis incidence. Our study revealed a positive genetic association between obesity in childhood and sepsis incidence. Firstly, Simmonds et al. have suggested that the likelihood of obesity in adulthood is five times higher in obese children compared to non-obese children (34). Our research also revealed an association between adult obesity and sepsis. Therefore, the correlation between childhood obesity and sepsis may be mediated by adult obesity. Secondly, previous research has firmly established childhood obesity as a risk factor for some health conditions, encompassing diabetes, essential hypertension, and Alzheimer’s Disease (35, 36). These conditions are recognized risk factors for sepsis and have been associated with elevated mortality rates in sepsis cases (1). It is probably that obesity in childhood heightens susceptibility to sepsis through the manifestation of these associated health issues. Thirdly, obesity in childhood may also increase the risk of sepsis through the gut microbiome-immune axis. Research has demonstrated an association between alterations in the gut microbiota and increased susceptibility to sepsis (37), with obesity causing shifts in the composition of the intestinal bacterial community (38). An expanding body of evidence suggests that the gut microbiota plays a pivotal role in immune system maturation and disease prevention throughout infancy, childhood, and adulthood. During early life, the body’s T cell pool expanse alongside the proliferation of human intestinal flora steadily. Intestinal dendritic cells in infancy consume colonizing bacteria in the gut, stimulating the development of bacteria-specific T cells (39). Gut dysbiosis can also lead to changes in hematopoietic stem cells in the bone marrow and alter the differentiation of progenitor cells, particularly in the context of obesity (40). The immune system, shaped by the early-life intestinal microbiota in obesity individuals, may be more susceptible to perturbation by pathogens and develop sepsis. In summary, prioritizing weight management in children may help prevent sepsis. With obesity increasingly affecting more children, maintaining a healthy weight during childhood becomes even more crucial.

In line with the findings of Hu et al., our study revealed a positive association between genetically predicted adult BMI and sepsis (41). Actually, BMI as an indicator of overall obesity cannot precisely capture the distribution and content of fat. The location of fat distribution is associated with susceptibility to specific diseases. For instance, individuals with abdominal obesity, as assessed by WC or visceral adiposity, are more prone to type 2 diabetes and adverse cardiovascular events (42). An 8-year longitudinal cohort study involving 30,239 subjects indicated that WC was a superior predictor of future sepsis risk compared to BMI (43). Although a higher BMI correlates with increased WC, visceral fat, and BFP, variations in the three indicates also exist among individuals with a normal BMI. Therefore, we investigated the relationship between WC, visceral adiposity, BFP, and susceptibility to sepsis, respectively. Our results suggested that genetically predicted WC and visceral adiposity were associated with an increased risk of sepsis, whereas genetically predicted adult BFP did not. There are some explanations for the causal relationship between adult adiposity and sepsis. First, obese individuals are more prone to develop skin folds and excessive sweating, which favor microbial proliferation and impede wound healing. Second, the susceptibility to infections associated with obesity may also be attributed to high blood sugar, hyperinsulinemia, and leptinemia, which lead to a weakening of both innate and adaptive immune responses (44). Third, adipose tissue has the capability to induce inflammation through its direct secretion of pro-inflammatory mediators and elevated levels of adipokines (45). Excessive fat accumulation can become a significant source of cytokine production in the body following an infection. Furthermore, the adipose tissue within the body can be categorized into visceral fat and subcutaneous fat. Research has demonstrated that, when compared to subcutaneous fat, visceral fat is more susceptible to lipolysis, leading to an elevation in free fatty acids, and an increasing the risk of metabolic syndrome. Visceral fat is also more prolific in the secretion of pro-inflammatory cytokines (46). This observation may partially explain the association between an elevated risk of sepsis and factors such as WC and visceral adipose, while BFP does not demonstrate a significant relationship. In conclusion, these results underscore the importance of not only monitoring BMI but also controlling WC and visceral fat content to combat susceptibility to sepsis.

This study boasts several notable strengths. First and foremost, we ventured to comprehensively explore the causal link between obesity and sepsis throughout the entire life cycle for the first time, utilizing GWAS data from newborn infants, children, and adults. This might be the first study confirm the association between obesity in childhood and subsequent sepsis. Second, the utilization of Mendelian randomization design mitigates biases, confounding factors, and reverse causality effects, rendering the conclusions more reliable. Last, the inclusion of a large sample size of GWAS data in this study substantially enhances statistical efficiency, allowing for a more robust result.

Our study has some limitations. Firstly, MR analysis operates on the premise of a linear relationship between exposure and outcome, which restricts our ability to assess non-linear associations using summary-level data. Underweight can also be a risk factor of sepsis (47). However, we were unable to analyze the relationship between underweight and sepsis. Due to data constraints, stratified analyses based on exposure factors were not feasible. Secondly, it remains uncertain whether our findings can be generalized to other racial and ethnic groups, although limiting our study to European populations helps mitigate population structure bias. Lastly, while sensitivity analysis confirms the robustness of the MR results, further validation is warranted through additional high-quality, population-based observational studies.

## Conclusion

In summary, our MR study has uncovered a noteworthy connection between childhood BMI, childhood obesity, adult BMI, adult WC, and adult visceral adiposity, and an increased incidence of sepsis. It’s important to recognize that, the issue of childhood obesity requires further attention. Additionally, even in individuals classified as ‘non-obese’ based on a normal BMI, increased WC or greater visceral fat should also be a cause for concern. Obesity and excess fat are modifiable risk factors that can be addressed through lifestyle interventions. It is crucial to educate the public about obesity and the various forms of fat accumulation.

## Data availability statement

The original contributions presented in the study are included in the article/**Supplementary Material**. Further inquiries can be directed to the corresponding authors.

## Ethics statement

Ethical approval was not required for the studies involving humans because this study was based on public databases and published studies, no additional ethics approval was needed. The studies were conducted in accordance with the local legislation and institutional requirements. Written informed consent for participation was not required from the participants or the participants’ legal guardians/next of kin in accordance with the national legislation and institutional requirements because this study was based on public databases and published studies, no additional informed consent was needed.

## Author contributions

ZC: Writing – review & editing, Writing – original draft, Visualization, Software, Methodology, Formal analysis, Data curation, Conceptualization. JL: Writing – review & editing, Writing – original draft, Visualization, Software, Methodology, Formal analysis, Data curation, Conceptualization. WT: Writing – review & editing, Writing – original draft, Visualization, Software, Methodology, Formal analysis, Data curation, Conceptualization. TL: Writing – review & editing, Supervision, Resources, Methodology, Conceptualization. CZ: Writing – review & editing, Supervision, Resources, Methodology, Conceptualization. JM: Writing – review & editing, Supervision, Methodology, Funding acquisition, Conceptualization. GL: Writing – review & editing, Supervision, Methodology, Funding acquisition, Conceptualization.

## Supplementary material

Supplementary Figure 1 MR leave-one-out sensitivity analysis for the effect of life course adiposity on sepsis.
