# Supplementary figures and images for: Exploring the relationship between life course adiposity and sepsis: insights from a two-sample Mendelian randomization analysis
Source: Front Endocrinol (Lausanne). 2024 Jun 14;15:1413690. doi: 10.3389/fendo.2024.1413690 (PMC11211544; doi:10.3389/fendo.2024.1413690)

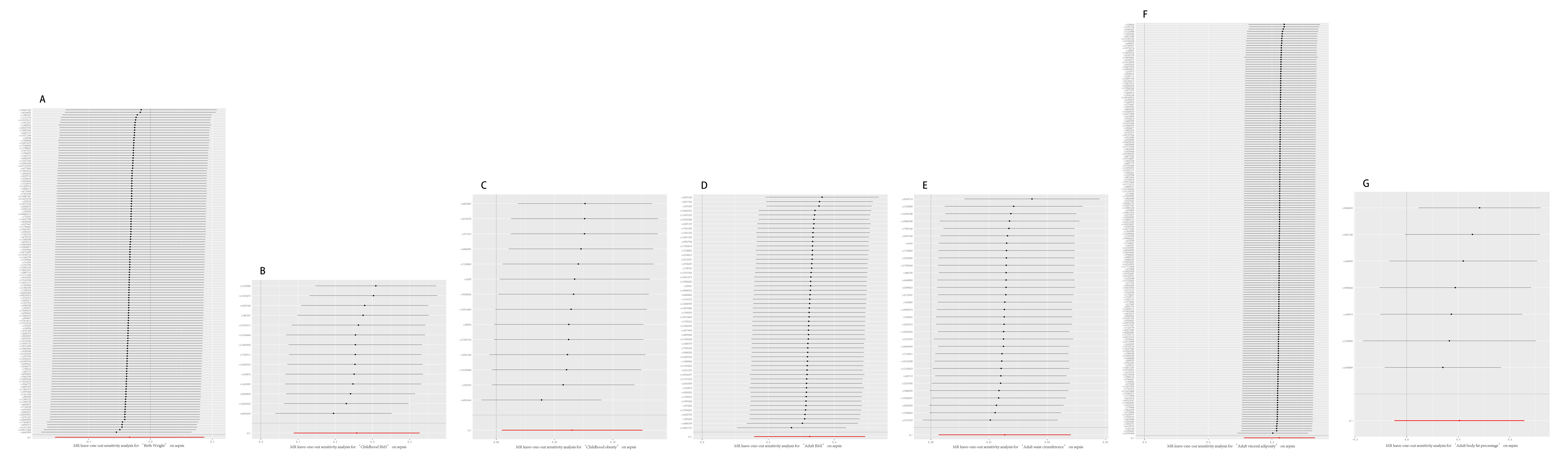

Supplement: Supplementary Figure 1 — MR leave-one-out sensitivity analysis for the effect of life course adiposity on sepsis. [file Image_1.jpeg]
